# A new T-type electrosurgical knife with waterjet function used in probe mode: a safe technical variant for colorectal endoscopic submucosal dissection

**DOI:** 10.1055/a-2336-0941

**Published:** 2024-06-25

**Authors:** Felipe Ramos-Zabala, Francisco J. Gallego Rojo, Julio Guilarte López-Mañas, Francisco Gallardo Sánchez, Sara Reina Serrado, Marian García-Mayor, Alejandra Alzina-Pérez

**Affiliations:** 1 Gastroenterology, HM Monteprincipe University Hospital, Madrid, Spain; 2 16345 Clinical Sciences, Faculty of Medicine, Universidad San Pablo CEU, Madrid, Spain; 3 16579 Gastroenterology, Hospital de Poniente de Almeria, El Ejido, Spain; 4 Gastroenterology, Hospital Comarcal de Baza, Baza, Spain

The water-jet hydrodissection technique is an effective method for colorectal endoscopic submucosal dissection (ESD) 1 , even during the technique learning curve 2 . The use of the T-type HybridKnife in “probe mode” can facilitate ESD 3 , even in complex situations 4 5 . The design of the new HybridKnife Flex ( Fig. 1 ) may improve the precision and safety of the technique.

**Fig. 1:**
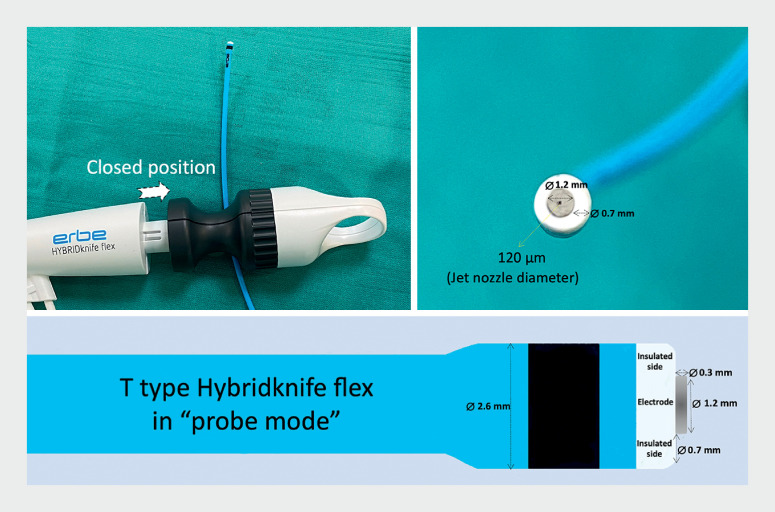
Photographs and illustration of the design features of the new T-type HybridKnife Flex in probe mode, with a 0.3-mm depth and 1.2-mm contact surface.

A 68-year-old woman was referred for ESD of a rectal polyp after screening colonoscopy. A 45-mm lesion, with granular nodular mixed laterally spreading tumor (LST) morphology, was found to be located 10 cm above the dentate line ( Fig. 2 ). Therapeutic endoscopy was performed using a T-type HybridKnife Flex of 1.5 mm, the ERBEJET system, a VIO3 electrosurgical unit (ERBE, Germany), and a colonoscope (Fujifilm, Japan) with transparent hood (Olympus, Japan) ( Video 1 ). In each phase of the ESD, we performed dynamic adjustment of the electrosurgical unit settings ( Fig. 3 ). The submucosal dissection was performed in preciseSECT mode with continuous activation (no “bumps”), allowing faster movement of the HybridKnife for stepwise dissection with enhanced hemostasis. The Flex design caliber is 2.6 mm and facilitates dissection with the endoscope being pushed without touching the knife, in a similar manner to painting on canvas (“brush technique”). The coagulation of blood vessels was carried out in probe mode using soft coagulation, approaching without any mechanical pressure, with continuous activation; vessels were subsequently cut with preciseSECT. The procedure time was 45 minutes. The resected specimen size was 65 × 50 mm ( Fig. 4 ). Histopathologic examination identified a tubular adenoma with intramucosal adenocarcinoma and free lateral and vertical resection margins.

**Fig. 2:**
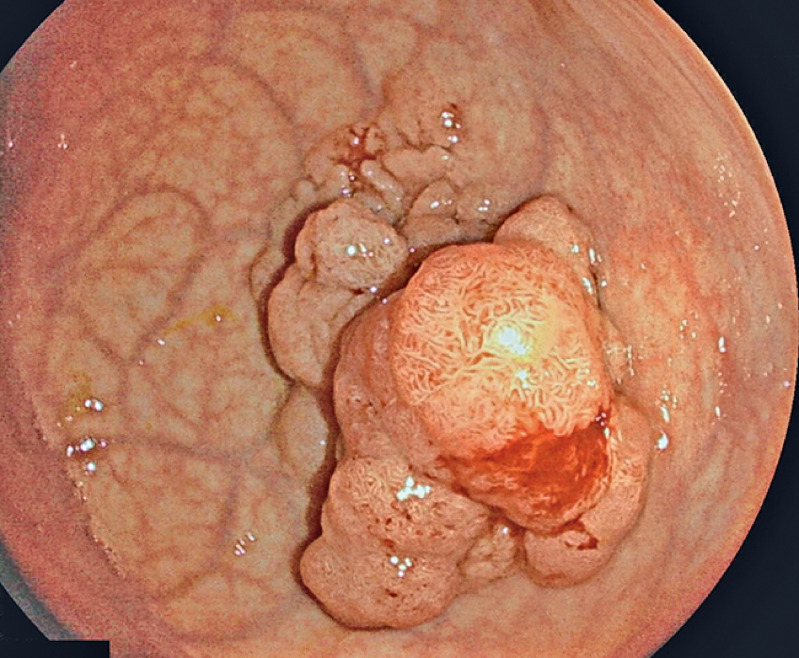
Endoscopic image showing a 45-mm lesion with granular nodular mixed laterally spreading tumor morphology that was located 10 cm above the dentate line.

**Fig. 3:**
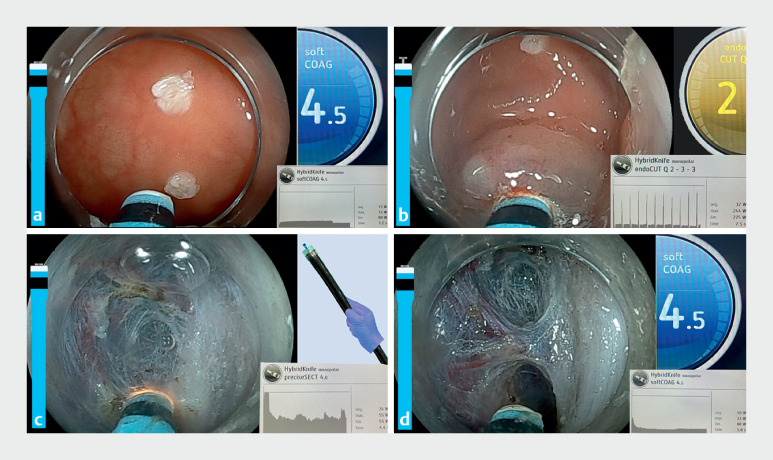
The dynamic adjustment of the electrosurgical unit settings is shown during the phases of endoscopic submucosal dissection: **a** thermocautery marks are made around the edge of the lesion using soft coagulation mode with the HybridKnife in probe mode; **b** to access the submucosa, endoCUT mode is used with the HybridKnife open to 1.5 mm; **c** the submucosal dissection phase is performed in preciseSECT mode with HybridKnife in probe mode (the Flex design has a diameter of 2.6 mm, which provides stability in the working channel of the endoscope, allowing dissection to be carried out by pushing the endoscope without having to touch the knife, in a manner similar to painting on canvas [“brush technique”]); **d** coagulation of blood vessels is carried out in probe mode using soft coagulation mode, with preciseSECT subsequently used to cut them.

**Fig. 4:**
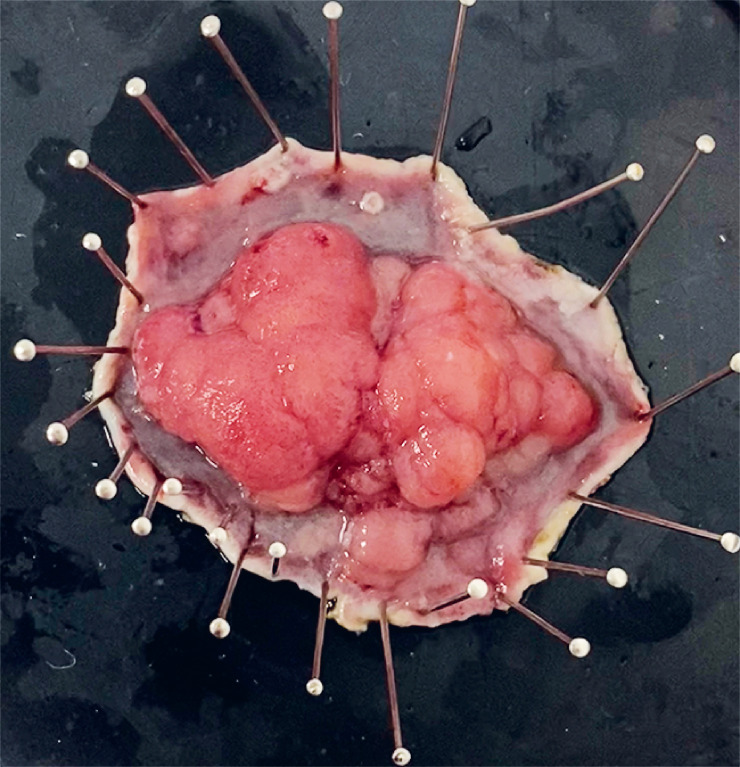
Macroscopic appearance of the resected specimen.

The new HybridKnife Flex is used for the removal of a rectal lesion. The submucosal dissection phase using preciseSECT mode with the HybridKnife in probe mode allows dissection to be performed using the “brush technique.”Video 1

The new HybridKnife Flex used in probe mode may be a promising alternative technique for ESD as it significantly facilitates the precision of the technique. The flexibility and finesse of the electrode in combination with the electrocautery settings of the VIO3 electrosurgical unit and water-jet hydrodissection technique could in future simplify ESD.

Endoscopy_UCTN_Code_TTT_1AQ_2AD_3AD

## Notes

Endoscopy E-Videos https://eref.thieme.de/e-videos
*E-Videos* is an open access online section of the journal *Endoscopy* , reporting on interesting cases and new techniques in gastroenterological endoscopy. All papers include a high-quality video and are published with a Creative Commons CC-BY license. Endoscopy E-Videos qualify for HINARI discounts and waivers and eligibility is automatically checked during the submission process. We grant 100% waivers to articles whose corresponding authors are based in Group A countries and 50% waivers to those who are based in Group B countries as classified by Research4Life (see: https://www.research4life.org/access/eligibility/ ). This section has its own submission website at https://mc.manuscriptcentral.com/e-videos .
